# CLEC14A was up-regulated in hepatocellular carcinoma and may function as a potential diagnostic biomarker

**DOI:** 10.1016/j.clinsp.2022.100029

**Published:** 2022-05-14

**Authors:** Lang Yan, Xiang Li, Yunfeng Yuan

**Affiliations:** Chongqing University Three Gorges Hospital, Chongqing, China

**Keywords:** CLEC14A, Hepatocellular carcinoma, ROC, Diagnosis

## Abstract

**Objective:**

The current work aimed to investigate the expression and potential clinical significance of C-type Lectin domain family 14 (CLEC14A) in hepatocellular carcinoma.

**Methods:**

The relative expressions of CLEC14A in the Hepatocellular Carcinoma (HCC) tissue and adjacent normal tissue of 105 HCC patients were examined using RT-qPCR methods. Furthermore, Receiver Operating Characteristic (ROC) curve was drawn for exploring the diagnostic value of CLEC14A. Next, the expressions of CLEC14A in HCC cell lines and normal liver epithelial cells were compared, and the effects of knockdown of CLEC14A on the growth and apoptosis of HCC cells were examined.

**Results:**

The authors found that the expression of CLEC14A was markedly increased in hepatocellular carcinoma tumors in comparison with the adjacent tissue, and the expression level of CLEC14A was positively correlated with the size and differentiation of the tumor. Moreover, results of ROC analysis showed CLEC14A might function as a sensitive diagnostic biomarker for HCC. Furthermore, CLEC14A was up-regulated in HCC cell lines, and transient over-expression of CLEC14A decreased the proliferation and increased the apoptosis of HCC cells *in vitro*.

**Conclusions:**

Our results suggested that CLEC14A was up-regulated in HCC and might function as a potential diagnostic marker.

## Highlights

• Over-expression of CLEC14A was in HCC.
• CLEC14A may function as a potential diagnostic biomarker for HCC.
• Over-expression of CLEC14A was in HCC cells.
• Knockdown of CLEC14A decreased the viability and increased the apoptosis of HuH-7 cell.

## Introduction

Hepatocellular Carcinoma (HCC) is a common malignancy of the liver system 1, 2, 3. Nowadays, because of the changes in the climate, lifestyle as well as structure of the diet, the incidence rate of HCC is increasing every year 4, 5, 6. The early stage of HCC did not have special symptoms; therefore, most of the HCC patients reached the advanced stage when HCC was first diagnosed. This always leads to the poor survival rate of the disease 7, 8, 9. Thus, to further explore the possible underlying mechanism of the genesis of HCC and find reliable biomarker is of great importance for the diagnosis and treatment of HCC.

In recent years, great efforts have been made to identify biomarkers for the early diagnosis of HCC. For example, Luo et al. identified and verified several new serum metabolite biomarkers which have shown good diagnostic value for HCC [10]. Moreover, by using mass spectrometry technology, Li et al. found that inter-alpha-trypsin inhibitor heavy chain four may be used for the early diagnosis of HCC [11]. Furthermore, Qin et al. suggested that the serum expression of ST8SIA6-AS1 may be a potential diagnostic biomarker for HCC [12].

The C-type Lectin-like receptors (CLECs) family consist of different transmembrane pattern recognition receptors 13, 14, 15. Previous studies suggested that CLECs play key roles in different biological events. For example, regulating the growth of cancer cells, maintaining body hemostasis, facilitating cell communication, etc 16, 17, 18. CLEC14A is a newly discovered CLEC protein, and reports on the roles of CLEC14A in different diseases were rate. Studies on CLEC14A were mainly focused on the regulator roles of CLEC14A in immune cells and angiogenesis 19, 20, 21.

The roles of CLEC14A in some types of cancers, for example, renal clear cell carcinoma [22] lung adenocarcinoma [23], uterine carcinosarcoma [24], have been discussed previously. However, up to now, the potential roles of CLEC14A in hepatocellular carcinoma still need to be explored. Therefore, the authors designed the present study to investigate the potential clinical value of CLEC14A in hepatocellular carcinoma.

## Materials and methods

### Sample collection and clinical information

In the present study, the authors enrolled a total of 105 patients with HCC. Patients were all pathological diagnosed with HCC by biopsy at Chongqing University Three Gorges Hospital. None of the patients received anti-tumor therapies before diagnosis. The HCC tumor samples, as well as the adjacent non-tumorous normal tissues (3‒5 cm from the tumor samples) of 105 patients were collected during the surgery process. The PBMCs of the patients and healthy controls were also collected. All tissue and serum samples were stored at -80°C until needed. The content of the current work has been approved by the ethical committee of Chongqing University Three Gorges Hospital (n° 500032175), and each patient signed the informed consent.

### Real-time quantitative polymerase chain reaction (RT-qPCR)

RNA of the liver tissues, PBMCs, and cells was extracted by TRIzol® reagent (Invitrogen; Thermo Fisher Scientific, Inc.). Reverse transcription and qPCR were carried out using One Step SuperRT-PCR Mix Kit (T2240; Solarbio) on Mastercycler® nexus (6330000072; Eppendorf, Inc.). All primers used in the present study were designed and synthesized by Genewiz Inc. GAPDH was used as the internal reference gene. The thermocycling conditions were as follows: denaturation at 94°C for 60s, then annealing at 37°C for 60s for 30 cycles. At last, the extension was at 72°C for 120s. The 2^ΔΔCt^ method was used for calculating the fold change in gene expression level. The sequences of the primers were: CLEC14A forward primer, 5’-CTGCACCACGCTACCATGAA-3’; CLEC14A reverse primer, 5’-CCAGGAGAAACCCCGCAAA-3’; GAPDH forward primer, 5’-TGTGGGCATCAATGGATTTGG-3’; GAPDH reverse primer, 5’- ACACCATGTATTCCGGGTCAAT-3’.

### Cell culture

Human normal liver cell line L02 cells, as well as HCC cell lines, including HuH-7 and SK-HEP-1 were purchased from the Cell Bank of China (Shanghai, China). The cells were maintained by PRMI-1640 medium containing 10% FBS as well as penicillin-streptomycin (Beyotime, Shanghai, China).

### Transfection

CLEC14A siRNA was obtained from Genepharma (Shanghai, China). To knock down CLEC14A expression in HuH-7 cells, the cells were transfected with 50 nM CLEC14A siRNA using Lipofectamine 3000 (Invitrogen) based on the protocols provided by the manufacturer.

### Cell viability assay

To determine the effects of CLEC14A on the viability of HCC cells, CCK-8 proliferation analysis was performed. Briefly, HuH-7 cells have been seeded onto 96-well plates (1 × 10^4^ cells/well) and transfected with or without CLEC14A siRNA. To detect the cell viability, each well was treated by CCK-8 reagent at a different time point, and the OD value at 450 nm was recorded by a microplate reader.

### Cell apoptosis assay

Annexin V/PI cell apoptosis kit was used to determine the effects of CLEC14A on the apoptosis of HuH-7 cells. Briefly, cells were maintained in a 6-well plate and treated with Annexin V and PI according to the instructions provided by the manufacturer. The apoptosis of the cells in each sample has been determined using a flow cytometer (FACSCalibur Flow Cytometer, BD, CA, USA).

### Statistical analysis

Statistical analysis has been carried out by SPSS 23.0 (Chicago, IL, USA). Data were shown as a mean ± SD. Comparison between two groups was analyzed by student *t*-test. Receiver Operating Characteristic (ROC) was used for evaluating the potential diagnostic value of CLEC14A. If the p-value is less than 0.05, the authors considered the comparison between the two groups were significantly different.

## Results

### Over-expression of CLEC14A was in HCC

**Fig. 1:**
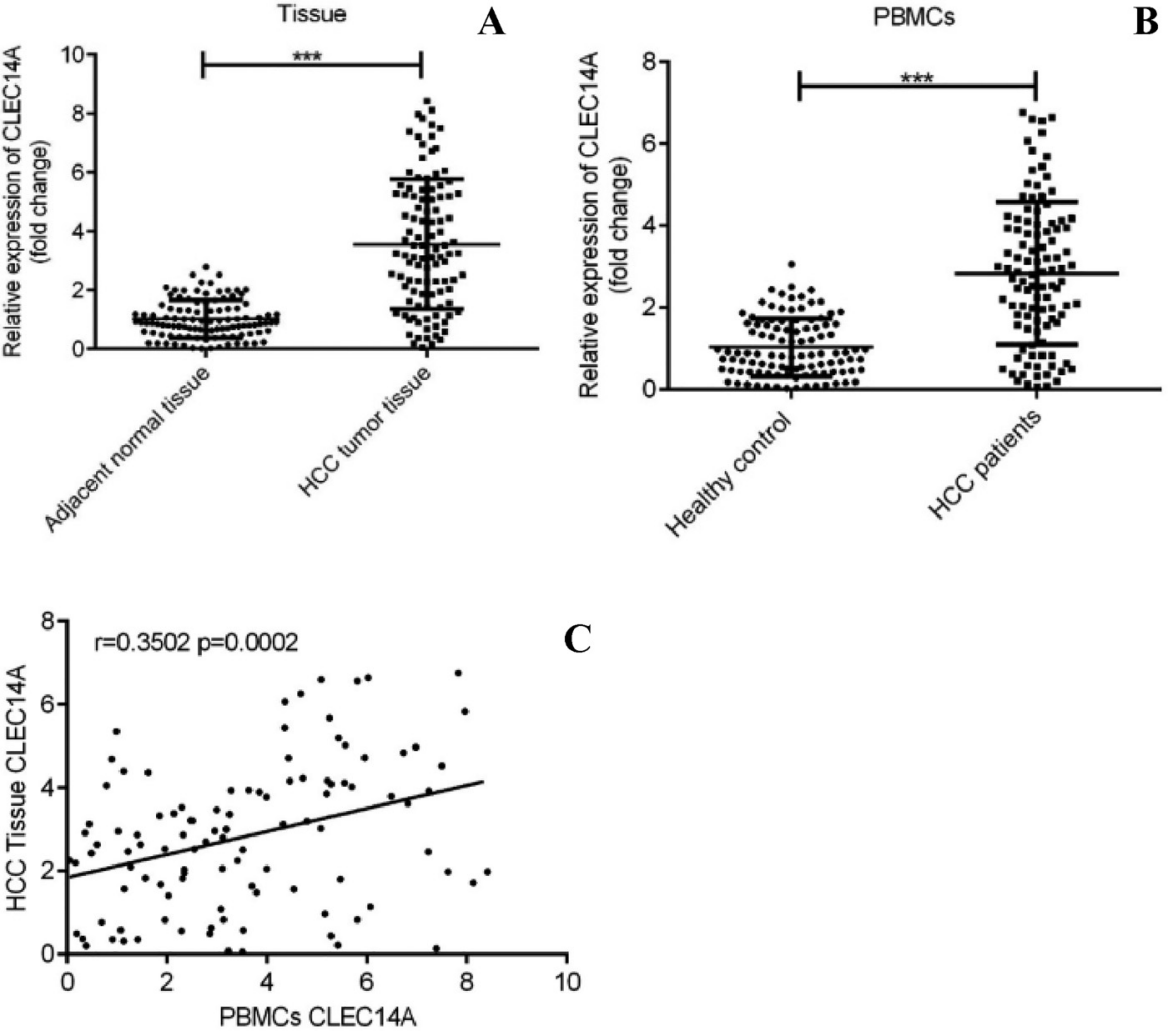
Up-regulation of CLEC14A in HCC. (A) Comparison between the expression of CLEC14A in HCC tumor and normal adjacent tissues. (B) Comparison between the expression of CLEC14A in PBMCs of HCC patients and healthy controls. (C) Results of correlation analysis. *** *p* < 0.01.

Firstly, the expression of CLEC14A in HCC tumor tissue, as well as non-tumorous adjacent tissues, was examined and compared. The clinical information of the patients is shown in Table 1. Increased expression of CLEC14A was correlated with tumor size (*p* = 0.0423) and differentiation (*p* = 0.0255). The authors found that CLEC14A mRNA expression was dramatically increased in HCC tumor tissues in comparison with the adjacent non-tumorous tissues (Fig. 1A, *p* < 0.01). Moreover, CLEC14A mRNA expression was also significantly increased in PBMCs of HCC patients (Fig. 1B, *p* < 0.01), and the levels of CLEC14A in HCC tumors and PBMCs were positively correlated (Fig. 1C; *r* = 0.3502, *p* = 0.0002).

**Table 1:** Clinicopathological characteristics of the patients.

	CLEC14A low group (*n* = 51)	CLEC14A high group (*n* = 54)	*p*-value
Age			0.6865
≥ 60	35	39	
< 60	16	15	
Sex			0.8049
Male	37	38	
Female	14	16	
Tumor size			**0.0423^a^**
< 5	26	17	
≥ 5	25	37	
TNM stage			0.1989
I-II	30	25	
III-IV	21	29	
Tumor nodules			0.6962
1	40	44	
≥ 2	11	10	
Cirrhosis			0.5237
Yes	22	20	
No	29	34	
Differentiation			**0.0255^a^**
Well-moderate	30	20	
Poor	21	34	
Metastasis			0.0985
Yes	22	32	
No	29	22	

*p < 0.05.

### CLEC14A may function as a potential diagnostic biomarker for HCC

**Fig. 2:**
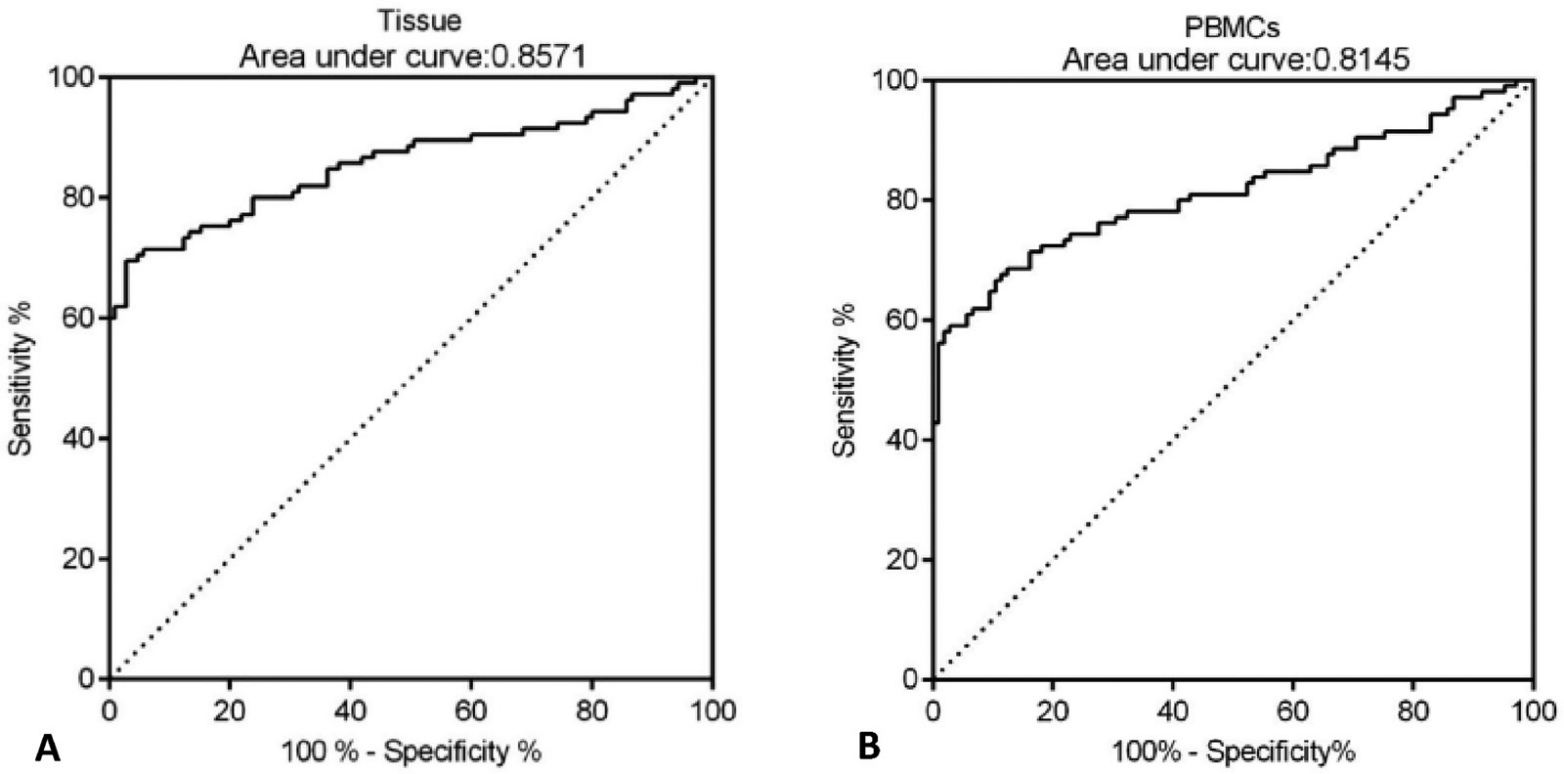
CLEC14A may function as a potential diagnostic biomarker. (A) Results of ROC analysis for tissue expression of CLEC14A. (B) Results of ROC analysis for PBMCs expression of CLEC14A.

Next, the potential diagnostic value of CLEC14A for HCC was determined by ROC analysis. The authors found that the AUC value of CLEC14A expression in tissue samples for the diagnosis of HCC was 0.8571 (Fig. 2A, 95% CI = 0.8032 to 0.9109), and for CLEC14A expression in PBMCs for the diagnosis of HCC was 0.8145 (Fig. 2B, 95% CI = 0.7542 to 0.8748).

### Over-expression of CLEC14A was in HCC cells

**Fig. 3:**
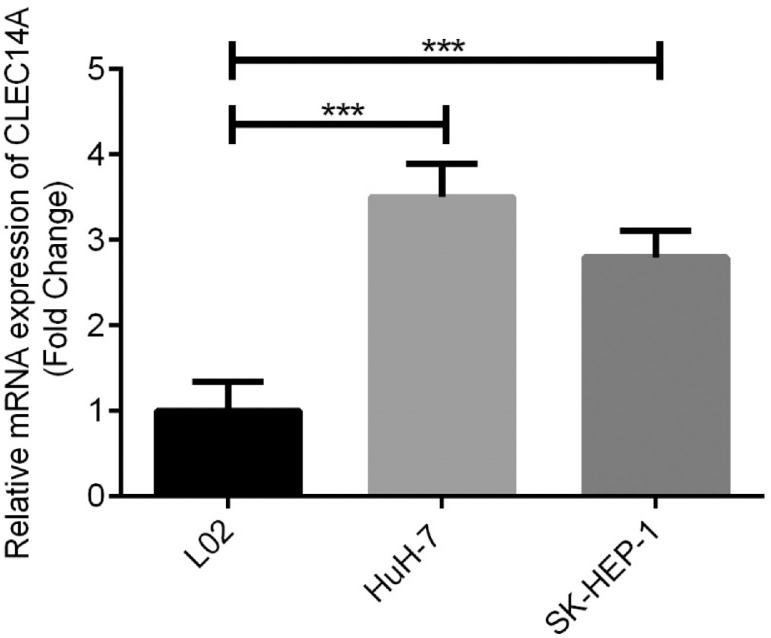
Up-regulation of CLEC14A in HCC cells. *** *p* < 0.01.

Furthermore, expressions of CLEC14A in human normal liver cell line L02 cells, HCC cell lines HuH-7 and SK-HEP-1 were examined and compared. The authors found that the expression of CLEC14A was significantly increased in both HuH-7 and SK-HEP-1 cells in comparison with L02 cells (Fig. 3, *p* < 0.01). Because HuH-7 has shown a higher CLEC14A level than SK-HEP-1, it has been used for further analysis.

### Knockdown of CLEC14A decreased the viability and increased the apoptosis of HuH-7 cell

**Fig. 4:**
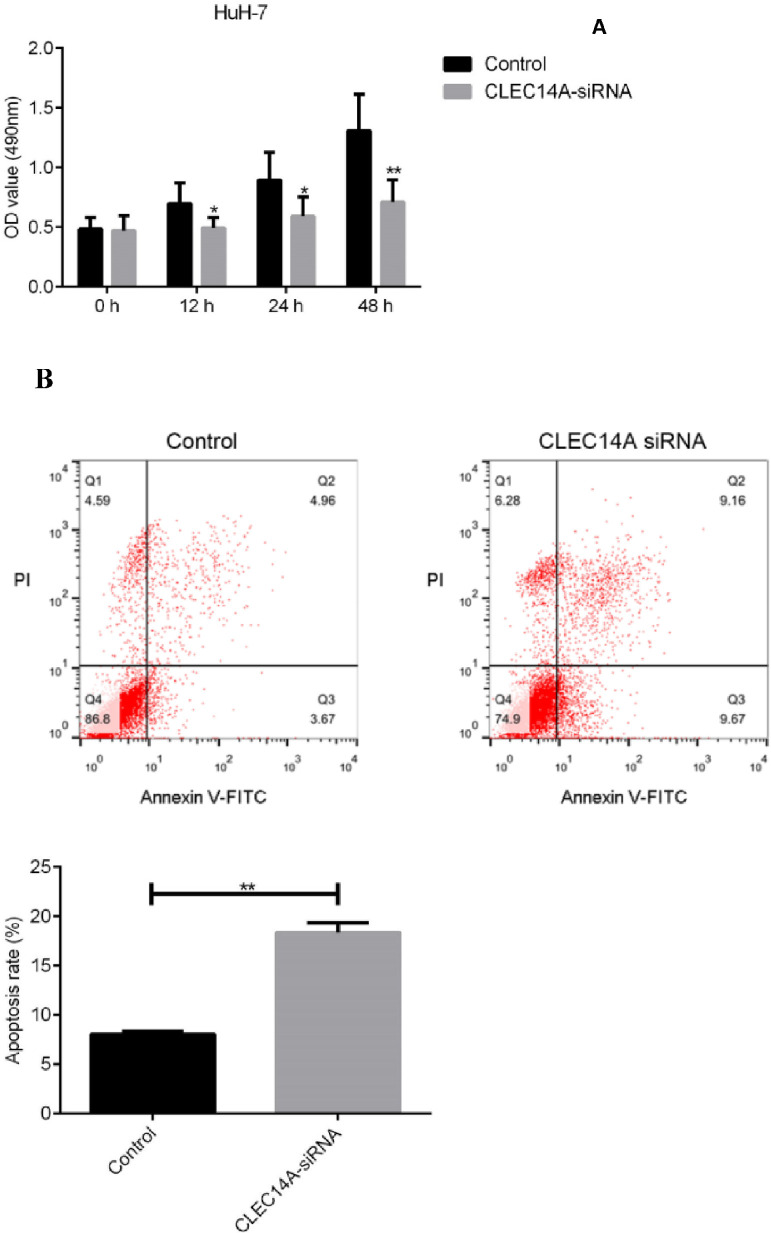
Effect of CLEC14A on the growth and apoptosis of HCC cells *in vitro*. (A) Effect of CLEC14A on the viability of HCC cells in vitro (** *p* < 0.01). (B) Effect of CLEC14A on the apoptosis of HCC cells in vitro (** *p* < 0.01).

Finally, to further investigate the roles of CLEC14A in the pathogenesis of HCC, the authors cultured HuH-7 cells with CLEC14A siRNA, and the effects of CLEC14A on the cell growth as well as apoptosis was determined by MTT and flow cytometry methods. Results of the MTT assay indicated that CLEC14A siRNA-transfected cells had shown decreased cell viability on 24h, 48h, and 72h (Fig. 4A). Moreover, down-regulation of CLEC14A also significantly promoted the apoptosis of HuH-7 cells *in vitro* (Fig. 4B, *p* < 0.01).

## Discussion

In the present study, the authors have explored the roles of CLEC14A in HCC. The present study's results proved that CLEC14A may function as an oncogene in HCC, and CLEC14A may exert its carcinogenic effects via increasing the viability and decreasing the apoptosis of HCC cells.

In recent years, CLEC family proteins were found to be up-regulated in several cancers, and the roles of CLEC14A as onco-genes have been investigated in different works [20,24,25], However, little was known about the function of CLEC14A in HCC. In the present study, the authors have observed that CLEC14A was significantly up-regulated in HCC tumors on both mRNA and protein levels; moreover, results of the clinical analysis showed the levels of CLEC14A in HCC patients were positively correlated with tumor size and differentiation. Interestingly, the results of ROC analysis (AUC 0.8571 for tissue and 0.8145 for PBMCs) indicated that the expression of CLEC14A can distinguish the tumor tissue and the non-tumor adjacent tissue. In a previous study, it has been reported that the AUC of Alpha-Fetoprotein (AFP), which is a currently used cancer biomarker for HCC, is 0.646, and for ITIH4, it is 0.667 [11]. Therefore, CLEC14A has shown better diagnostic value than the above biomarkers for the diagnosis of HCC. Taken together, the present study's data suggested that CLEC14A may function as an oncogene in HCC, and the levels of CLEC14A were positively correlated with the severity and progression of HCC.

The CLEC family proteins have been known to play critical roles in the proliferation and apoptosis of cancer cells [21,26,27]. To further investigate the roles of CLEC14A in the pathogenesis of HCC, the authors cultured HCC cells with CLEC14A siRNA, and the effects of CLEC14A on the viability and apoptosis of HuH-7 cells were examined. The authors observed that CLEC14A siRNA-transfected cells had shown decreased cell viability as well as increased apoptosis, suggesting that CLEC14A can regulate the growth and apoptosis of HCC cells *in vitro*.

The present study has several limitations. The authors only performed clinical analysis and cell studies, and in the future, the roles of CLEC14A in HCC should also be further explored using animal models. Also, current work was based on the Asian population, and the roles of CLEC14A in other races should also be evaluated.

In conclusion, the authors proved that CLEC14A was up-regulated in HCC, and CLEC14A can regulate the growth as well as apoptosis of HCC cells in vitro. The present data have provided novel evidence for the treatment of HCC.

### Authors' contributions

Lang Yan was responsible for the organization and coordination of the trial. Xiang Li was the chief investigator and was responsible for the data analysis. Lang Yan, Xiang Li, and Yunfeng Yuan developed the trial design. All authors contributed to the writing of the final manuscript.

## Conflicts of interest

The authors declare no conflicts of interest.

## References

[1] Kim TH, Woo S, Joo I, Bashir MR, Park MS, Burke LMB (2021). LI-RADS treatment response algorithm for detecting incomplete necrosis in hepatocellular carcinoma after locoregional treatment: a systematic review and meta-analysis using individual patient data. Abdom Radiol (NY).

[2] Li L, Xie R, Lu G. (2021). Identification of m6A methyltransferase-related lncRNA signature for predicting immunotherapy and prognosis in patients with hepatocellular carcinoma. Biosci Rep.

[3] Orzechowska D, Klimowicz K, Stepien A, Mikula T, Sapula M (2021). Wiercinska-Drapalo A. Change in ɣ-glutamyl transpeptidase activity as a useful tool in identifying a group of patients with elevated risk of hepatocellular carcinoma development after DAA treatment of chronic hepatitis C. Clin Exp Hepatol.

[4] Lapinski TW, Tarasik A, Januszkiewicz M, Flisiak R. (2021). Clinical aspects and treatment of hepatocellular carcinoma in north-eastern Poland. Clin Exp Hepatol.

[5] Nassar ES, Elkalbashawy YA, Kamal A, Zakaria NHE. (2021). Galectin-3 is not useful for hepatocellular carcinoma surveillance in cirrhotic patients, but it may be a marker of cirrhosis development. Clin Exp Hepatol.

[6] Kamal A, Elmoety AAA, Rostom YA, Shater MS, Lashen SA. (2021). Hepatocellular carcinoma recurrence after directly acting antivirals for chronic hepatitis C: a 2-year follow-up study. Clin Exp Hepatol.

[7] Wang H, Qiu W. (2021). EPHA2, a promising therapeutic target for hepatocellular carcinoma. Mol Cell Oncol.

[8] Wen L, Weng S, Yan C, Ye R, Zhu Y, Zhou L (2021). A radiomics nomogram for preoperative prediction of early recurrence of small hepatocellular carcinoma after surgical resection or radiofrequency Ablation. Front Oncol.

[9] Pan Y, Xia S, Cai J, Chen K, Cai X. (2021). Efficacy of laparoscopic hepatectomy versus open surgery for hepatocellular carcinoma with cirrhosis: a meta-analysis of case-matched studies. Front Oncol.

[10] Luo P, Yin P, Hua R, Tan Y, Li Z, Qiu G (2018). A Large-scale, multicenter serum metabolite biomarker identification study for the early detection of hepatocellular carcinoma. Hepatology.

[11] Li X, Li B, Li B, Guo T, Sun Z, Li X (2018). ITIH4: effective serum marker, early warning and diagnosis, hepatocellular carcinoma. Pathol Oncol Res.

[12] Qin SJ, Zhou HZ, Xu NS, Yang HC, Chen PX. (2020). The diagnostic value of serum ST8SIA6-AS1 as biomarker in hepatocellular carcinoma. Clin Lab.

[13] Oishi S, Tsukiji N, Otake S, Oishi N, Sasaki T, Shirai T (2021). Heme activates platelets and exacerbates rhabdomyolysis-induced acute kidney injury via CLEC-2 and GPVI/FcRɣ. Blood Adv.

[14] Martin EM, Zuidscherwoude M, Morán LA, Di Y, Garcia A, Watson SP. (2021). The structure of CLEC-2: mechanisms of dimerization and higher-order clustering. Platelets.

[15] Otake S, Sasaki T, Shirai T, Tsukiji N, Tamura S, Takano K (2021). CLEC-2 stimulates IGF-1 secretion from podoplanin-positive stromal cells and positively regulates erythropoiesis in mice. J Thromb Haemost.

[16] Meng D, Ma X, Li H, Wu X, Cao Y, Miao Z (2021). A role of the podoplanin-CLEC-2 axis in promoting inflammatory response after ischemic stroke in mice. Neurotox Res.

[17] Etemad M, Christodoulou F, Weiss C, Kluter H, Bugert P. (2021). Correlation of CLEC1B haplotypes with plasma levels of soluble CLEC-2 in healthy individuals. Platelets.

[18] Suzuki-Inoue K, Tsukiji N. (2020). Platelet CLEC-2 and lung development. Res Pract Thromb Haemost.

[19] Jang J, Kim MR, Kim TK, Lee WR, Kim JH, Heo K (2017). CLEC14a-HSP70-1A interaction regulates HSP70-1A-induced angiogenesis. Sci Rep.

[20] Mura M, Swain RK, Zhuang X, Vorschmitt H, Reynolds G, Durant S (2012). Identification and angiogenic role of the novel tumor endothelial marker CLEC14A. Oncogene.

[21] Rho SS, Choi HJ, Min JK, Lee HW, Park H, Park H (2011). Clec14a is specifically expressed in endothelial cells and mediates cell to cell adhesion. Biochem Biophys Res Commun.

[22] Robinson J, Whitworth K, Jinks E, Nagy Z, Bicknell R, Lee SP. (2020). An evaluation of the tumour endothelial marker CLEC14A as a therapeutic target in solid tumours. J Pathol Clin Res.

[23] Su C, Shi K, Cheng X, Han Y, Li Y, Yu D (2019). Methylation of CLEC14A is associated with its expression and lung adenocarcinoma progression. J Cell Physiol.

[24] Li J, Xing X, Li D, Zhang B, Mutch DG, Hagemann IS (2017). Whole-genome DNA methylation profiling identifies epigenetic signatures of uterine carcinosarcoma. Neoplasia.

[25] Kim TK, Park CS, Jang J, Kim MR, Na HJ, Lee K (2018). Inhibition of VEGF-dependent angiogenesis and tumor angiogenesis by an optimized antibody targeting CLEC14a. Mol Oncol.

[26] Zhuang X, Maione F, Robinson J, Bentley M, Kaul B, Whitworth K (2020). CAR T cells targeting tumor endothelial marker CLEC14A inhibit tumor growth. JCI Insight.

[27] Marg A, Escobar H, Karaiskos N, Grunwald SA, Metzler E, Kieshauer J (2019). Human muscle-derived CLEC14A-positive cells regenerate muscle independent of PAX7. Nat Commun.

